# Nutritional Status in Patients with Esophageal Cancer Receiving Chemoradiation and Assessing the Efficacy of Usual Care for Nutritional Managements

**DOI:** 10.31557/APJCP.2020.21.8.2315

**Published:** 2020-08

**Authors:** Sara Movahed, Abdolreza Norouzy, Ali Ghanbari Motlagh, Saeid Eslami, Majid Khadem-Rezaiyan, Maryam Emadzadeh, Mohsen Nematy, Majid Ghayour-Mobarhan, Fatemeh Varshoee Tabrizi, Federico Bozzetti, Mehdi Seilanian Toussi

**Affiliations:** 1 *Department of Nutrition, Faculty of Medicine, Mashhad University of Medical Sciences, Mashhad, Iran. *; 2 *Cancer Office, Deputy of Health, Ministry of Health, Tehran, Iran. *; 3 *Department of Medical Informatics, Faculty of Medicine, Mashhad University of Medical Sciences, Mashhad, Iran. *; 4 *Clinical Research Development Unit, Faculty of Medicine, Mashhad University of Medical Sciences, Mashhad, Iran. *; 5 *Clinical Research Unit, Faculty of Medicine, Mashhad University of Medical Sciences, Mashhad, Iran.*; 6 *Metabolic Syndrome Research Center, Faculty of Medicine, Mashhad University of Medical Sciences, Mashhad, Iran. *; 7 *Reza Radiotherapy and Oncology Center, Mashhad, Iran. *; 8 *Department of surgery, Faculty of Medicine, University of Milan, Lombardia, Italy.*; 9 *Cancer Research Center, Faculty of Medicine, Mashhad University of Medical Sciences, Mashhad, Iran. *

**Keywords:** Esophageal cancers, nutritional assessment, nutritional status, PG-SGA, chemoradiation

## Abstract

**Background:**

Malnutrition is prevalent in esophageal cancer patients which affects cancer prognosis. The purpose of this study was a comprehensive assessment of nutritional status during Chemoradiation (CRT).

**Methods:**

Newly diagnosed adults with esophageal cancer were recruited for this study. Patient-Generated- Subjective Global Assessment (PG-SGA), anthropometric indices, body composition, dietary intake, laboratory tests, and nutritional-related complications were assessed before, after, and 4 to 6 weeks after CRT.

**Results:**

Seventy-one cases were enrolled. The mean age was 66.8±12 years. Patients’ mean weight loss was 2.42±2.4 kilograms during treatment. A significant reduction observed in mean MUAC (26.68±4.9 vs. 25.42±5.1 cm), fat mass percentage (24.11±11.8 vs. 22.8±12.5), fat free mass index (16.87±2.4 vs. 16.47±2.6 kg/m^2^) and hand grip strength (43.2±19 vs. 36.1±20 kg) during CRT (all p-values <0.0001). We had also a non-significant change in mean energy intake (19.5±11 vs. 18.3±11 kcal/kgw. day) and protein intake (0.56±0.4 vs. 0.66±0.5 g/kgw.day) during CRT. In our assessment before, immediately after and 4-6 weeks following CRT, we recorded energy intake insufficiency in 55.7%, 58.7% and 27.3% and protein intake inadequacy in 89.8%, 89.1% and 72.7% of cases, respectively. The most common complications were dysphagia (56.7%), anorexia (25%), and constipation (47.9%) at admission. Dysphagia improved in some cases (42%), but anorexia (35%), early satiety (25%), Esophagitis (25%), dysosmia (21%) and dysgeusia (17%) were increased as CRT complication. yet, 25% of patients had dysphagia and 34.4% had constipation 4-6 weeks after CRT. The twelve-months mortality was significantly associated with lower BMI after CRT, primary PG-SGA score, weight loss, BMI<18.5, MUAC, physical performance, living in rural or urban areas, addiction.

**Conclusion:**

Our study demonstrated a high prevalence of malnutrition among esophageal cancer patients which worsened during Chemoradiotherapy. Our findings warrant early screening and monitoring of nutritional status and effective nutritional interventions with symptoms management during treatment in these patients.

## Introduction

Esophageal cancer (EC) usually has an aggressive nature with a 5-year survival rate of less than 20 %. EC is the eighth most prevalent cancer and the sixth cause of cancer death in the world (Enzinger and Mayer, 2003; Mao et al., 2011; Zhang, 2013). It is projected that EC prevalence will be increased unlike some other cancers (Lambert and Hainaut, 2007). The highest incidence of EC has been reported up to 100 cases per 100,000 annually in Asian population, China and the Middle East (Eslick, 2009). EC accounts for 9% of all types of cancers in Iran and 27% of gastrointestinal cancers (Ghavamzadeh et al., 2001; Salehiniya et al., 2018).

The incidence of malnutrition in upper gastrointestinal cancer patients is high affecting up to 90% of cases (Silvers et al., 2014). Weight loss and malnutrition is prevalent in patients with esophageal cancer (Anandavadivelan and Lagergren, 2016). The negative effects of malnutrition on treatment response, the quality of life and survival have been shown in previous trial. Moreover, malnutrition increases disease burden, costs, length of hospital stay and the probability of treatment discontinuation (Dewys et al., 1980; de Luis et al., 2006; Okamoto et al., 2018). Malnutrition may lead to cachexia which is responsible for up to 20 percent of cancer deaths (Gullett et al., 2011; Ryan et al., 2016). Cancer treatments including chemotherapy, radiotherapy and surgery can cause weight loss and reduced body mass index, muscle mass, and appetite (Anandavadivelan and Lagergren, 2016). Also, the importance of nutritional assessment before surgery has been demonstrated by several guidelines (Arends et al., 2017; Weimann et al., 2017; Low et al., 2019). Thus, nutritional assessment as the first step for early intervention is necessary for cancer patients. There are limited studies about nutritional status of esophageal cancer patients during chemoradiation (CRT).

The purpose of this observational study was a comprehensive assessment of nutritional status in esophageal cancer patients before, immediately after and 4 to 6 weeks after cancer treatments including radiotherapy (RT) or chemoradiotherapy (CRT) and to evaluate the importance of routine consultation with a nutritionist in these patients. 

## Materials and Methods

In this cross-sectional survey study, we recruited consecutive newly diagnosed patients over 18 with pathologically proven esophageal cancer who were referred for RT or CRT to Reza Radiation Oncology Center (RROC), Mashhad, Iran, from February 2017 to February 2018. This study is approved by the research ethics committee at Mashhad University of Medical Sciences (code: IR.MUMS.fm.REC.1395.111). An informed written consent was obtained from all participants. 

The demographic, economic status (Payab et al., 2012), disease related, and the treatments data were obtained through interviews and medical notes. Nutritional assessment data were also collected before, immediately after CRT and 4 to 6 weeks following CRT. 

In this observational study our goal was to evaluate the efficacy of current approach for nutritional management in patients with cancer. Therefore, we did not practice active nutritional intervention. However, patients received general nutritional advices at the beginning of treatment by a dietician and were allowed to receive nutritional supplements or medication for relieving symptoms such as nausea as prescribed by their physician based on their own judgment. Metastatic patients who were a candidate for palliative treatment were not included. In this observational study, severely malnourished cases who needed active nutritional intervention were excluded in any part of follow-up. 

Weight was measured by using a calibrated (Seca 510 scale- Germany). The patient was asked to stand at the center of scale base, barefoot and wearing light clothes. BMI was calculated by dividing the patients’ weight (in kilograms) by the square of their height (in meters). To measure height, a wall mounted stadiometer was used (Seca 206 stadiometer, Germany). Weight loss (WL) was defined as either no weight loss (<2% of body weight), mild (2-5% loss in 1 month or 2-10% in 6 months), moderate (5-10% loss in 1 month or 10-20% in 6 months), and severe (>10% loss in 1 month or >20% in 6 months). Mid upper arm circumference (MUAC) was also measured by a flexible tape. The patients were told to bend the left arm and marked the mid-point between olecranon and acromion process with a pen. Then, the tape was wrapped around the marked point with hanging straight arm and MUAC was measured. Body composition was measured using bioelectric impedance analysis (Tanita BC-418, Japan) including fat mass and fat free mass (in kilogram and percentage). Fat free mass index (FFMI) was calculated as fat free mass (in kilogram) divided into the square of the height (in meter). FFMI of less than 17 in men and 15 in women is considered as reduced muscle mass (Cederholm et al., 2019).

 A nutrition specialist assessed the nutritional status on the basis of Patient Generated- Subjective Global Assessment (PG-SGA) which evaluates recent weight loss, patients’ intake, nutrition related complications, physical performance, nutrition impact diseases, fever, corticosteroid usage, muscle and fat wasting and edema (Bauer et al., 2002). PG-SGA is validated in Persian (Khodabakhshi et al., 2018; Shahabbasi J et al., 2018). Assessing physical condition was by means of physical performance status and muscle strength (Flood et al., 2014; Russell, 2015). We used Karnofsky performance status scale which is a validated tool for physical performance evaluation.(Schag et al., 1984). Hand grip strength measured with dynamometer (Jamar, USA).

We used visual analogue scale (VAS) for pain the patients was experiencing in any cite of the body (Williamson and Hoggart, 2005). For dysphagia score, 5 score tool was used which is explained as 0, No dysphagia: able to eat normal diet; 1, Moderate passage: able to eat some solid foods; 2, Poor passage: able to eat semi-solid foods; 3, Very poor passage: able to swallow liquids only; and 4, No passage: unable to swallow anything (Homs et al., 2004).

Patients’ dietary intake was recorded using a 24-hour food recall. Macro and micronutrients were assessed by Nutritionist 4 software version 7(N-squared computing, USA). Energy and protein requirements calculation was based on guidelines by the European Society of Parenteral and Enteral Nutrition (ESPEN): 30 kilocalories per kilogram of body weight per day and 1.2 grams per kilogram of weight per day for protein requirement. Energy insufficiency was defined as intake of less than 60 percent of their needs (Arends et al., 2017). Patients were also asked about food availability. The patients were asked if they can prepare enough food for themselves including shopping, cooking, and eating. If not, they were asked if they had someone to prepare food for them. 

After inclusion and filling the questionnaire, the patients were referred to laboratory fasted for 12 hours before sampling. total protein, and serum albumin measured by autoanalyzer device (Alpha classic- AT plus). quantitative C-reactive protein (CRP) measured by ELISA test. Complete blood count (CBC) measured by Sysmex KX-21N (Japan). 

RT completion and delay were also asked. RT delay was considered if the RT interruption were more than 7 days. The reason could due to technical problem such as radiotherapy accelerator breakdown or disease-related problem such as hospitalization or severe neutropenia. The twelve-months mortality was also followed.

Sample size calculation was according to usual care group in Isering et al. (2004) study according to PG-SGA score at the beginning and end of study considering r=0.4, α=0.05 and β=0.2 (Isenring et al., 2004). The sample size was calculated 30 subjects at the end of CRT and with a drop rate of 5% the final sample size should be at least 33 patients. All the data collected and analyzed in SPSS V.16 (IBM, USA). We used paired T-test and Chi square test for quantitative and qualitative variables, respectively. Significance level was considered as p-value less than 0.05.

## Results

**Figure 1:**
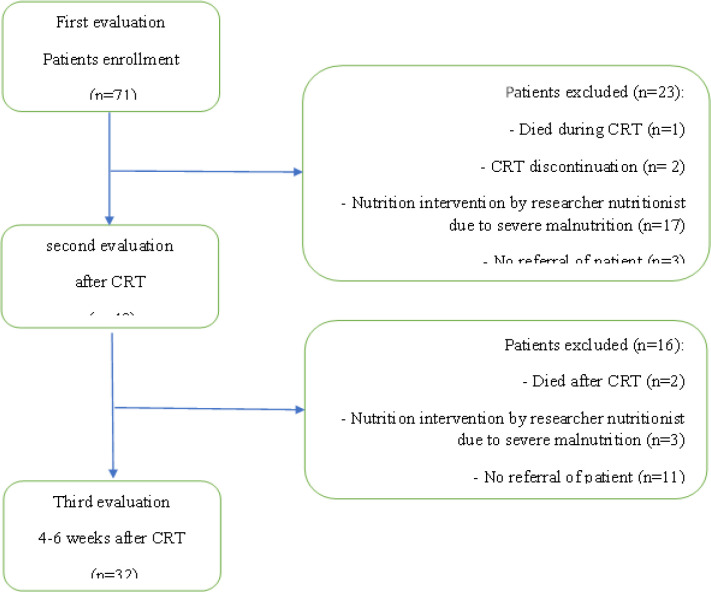
Flow-Diagram of Included Patients

 A total of 71 patients were enrolled in this study. Flow diagram of included patients is presented in Figure 1. The mean age was 66.3±12 years with the range of 35 to 87 years. Thirty-three patients (46.5%) were men. While 61% lived in rural areas, 6% were living alone (Table 1). Table 2 represents the tumor and treatment characteristics of the study participants. Overall, 64 patients (90.1%) were diagnosed as squamous cell carcinoma (SCC). The most prevalent tumor site was lower esophagus (50.7%). Patients received a median radiotherapy dose of 50.40 Gray (range 40 to 71 Gray). The common chemotherapy regimen was weekly cisplatin and weekly paclitaxel/carboplatin. 15 patients (21.1%) underwent surgery after CRT. 

Dietary intakes components are shown in table 3. The energy intakes were 19.5±11 and 18.3±11 kilo calories per kg of patients’ weight per day and protein intakes were 0.56±0.4 and 0.66±0.5 gram per kg of patients’ weight per day at baseline and after CRT. Dietary intakes of micronutrients were insufficient at baseline and after CRT, particularly vitamins D, E, zinc, iron, calcium, as well. Only vitamin C intake was sufficient in participants.

**Figure 2:**
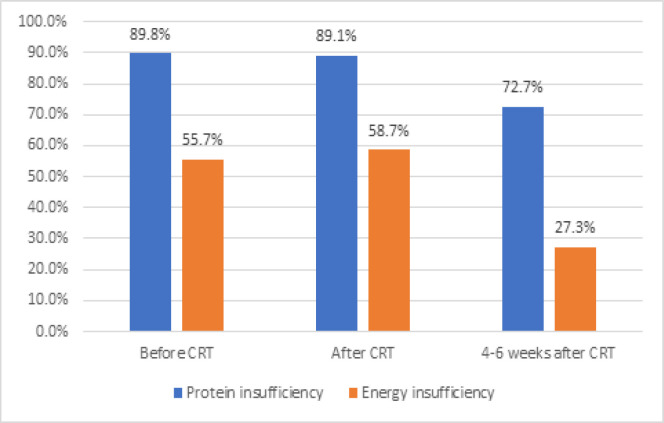
Energy and Protein Insufficiency Percentage in Participants During Chemoradiation

Figure 2 represents the energy and protein intake insufficiency percentage in participants during CRT according to ESPEN guidelines on nutrition in patients. Based on our assessment before, immediately after and 4 to 6 weeks post-treatment, we found energy intake insufficiency in 55.7%, 58.7% and 27.3% of patients and inadequate protein intake in 89.8%, 89.1% and 72.7% of patients, respectively. 

PG-SGA score, anthropometric indices, physical performance and laboratory tests in study participants are shown in table 4. According to PG-SGA scoring, 60 participants had PG-SGA>8 requiring nutritional interventions and symptom management and in 9 Patients (12.7%) with PG-SGA score of 4-8 needed dietary modification. 25 patients (35.2%) were underweight (BMI<18.5) and 30 cases (42.3%) had reduced sex adjusted muscle mass at the beginning of CRT. Besides, mild, moderate and severe weight loss was recorded in 26.7%, 32.4%, and 18.3% of patients during the treatment respectively. 

Nutritional status markers had decreased during CRT including white blood cells (WBC), total lymphocyte count (TLC), hemoglobin, red blood cell distribution width (RDW), serum total protein and albumin (p-value<0.0001).

**Figure 3:**
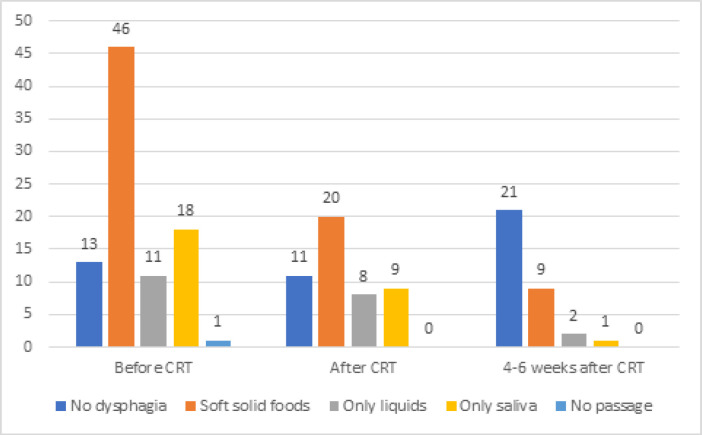
Dysphagia Intensity Frequency in Participants during Chemoradiation

The most common symptoms which led to dietary intake reduction were dysphagia (57%), constipation (48%), and anorexia (25%) at the beginning of CRT. After CRT, dysphagia was reported in 42% of patients. The other complications leading to food intake reduction were constipation (40%), anorexia (35%), early satiety (25%), esophagitis (25%), dysosmia (21%) and dysgeusia (17%). Four to six weeks after CRT, 25% of patients had dysphagia. The other food intake reducing complications were constipation (34%), esophagitis (27%), dyspepsia (27%), and anorexia (13%). Dysphagia scoring during chemoradiation in participants is shown in figure 3.

Oral nutrition supplementation was administrated by oncologists in 16 (22.5%) out of 48 patients who were assessed after treatment. Only 2 patients, oral supplementation was at least 200 kcal/day. It is worth reminding, these are the patients who did not visit dietician for nutritional intervention. Seventeen patients referred to researcher nutritionist during CRT and nutritional interventions were administrated for them and patients were monitored. The other 10 patients received intralipid or amino-acids serums in which none of the patients achieved to their energy or protein goals. 

With a median (25th -75th percentile) follow up time of 18 months (9 -23) months, 37 (52%) deaths were recorded. 21 patients (29.6%) died during the first year of follow up. The twelve-month mortality was significantly associated with BMI<18.5 before treatment (52% vs. 26%) (p-value=0.032). Also, there was a higher baseline PG-SGA score in deceased patients (16.5±6 vs. 12.4±5) (p-value= 0.005). We also found a significant association between twelve-month mortality and lower BMI after CRT (18.63±4.9 vs. 22.9±6.1) (p-value=0.022), lower baseline MUAC (p-value=0.013), lower baseline Karnofsky physical performance (p-value=0.012), more weight loss before CRT (p-value=0.013), living in rural areas (p-value=0.023), addiction (p-value=0.013), and low economic status (p-value=0.033) (Table 5).

**Table 1:** General Characteristics of Study Participants (n=71)

Variables	Frequency (%)
Age (years): Mean ± standard deviation	66.3±12
Sex (	
Male	33 (46.5)
Education	
Illiterate	48 (67.6)
Primary education	22 (31)
University education	1 (1.4)
Living area	
Rural	43 (60.6)
Marital status	
Married	54 (76.1)
Solitary living	4 (5.6)
Occupation	
House wife	21 (29.6)
Farmer	19 (26.8)
Worker	6 (8.5)
Self employed	1 (1.4)
Employee	4 (5.6)
Retired	20 (28.2)
Economic status	
Low	52 (73.2)
Moderate	14 (19.7)
High	5 (7)
Insurance support	
Without insurance support	4 (5.6)
Primary insurance	49 (69)
Complementary insurance	18 (25.4)
Food availability	
No need for assistance	44 (62)
Has somebody for food preparation	26 (36.6)
Needs help but nobody is available	1 (1.4)
Current smoking	18 (25.4)
Opium addiction	20 (28.2)
Cancer self-awareness	24 (33.8)
Medical history	
Diabetes	5 (7)
Cardiovascular diseases	7 (9.9)
Hypertension	22 (31)
Hyperlipidemia	8 (11.3)

**Table 2:** Tumor and Treatment Related Characteristics of Study Participants

Variables	Frequency (Percentage)
Diagnosis duration (months): Mean ± standard deviation	1.3±1
Tumor sites	
Upper	8 (11.3)
Middle	27 (38)
Lower	36 (50.7)
Tumor pathology	
Squamous cell carcinoma (SCC)	64 (90.1)
Adenocarcinoma	5 (7)
Undifferentiated	2 (2.9)
Treatment type	
Radiotherapy	9 (12.7)
Chemoradiation	62 (87.3)
Radiotherapy total dosage (Grays)	53.65±650#
Radiotherapy completion	66 (93)
Radiotherapy Delay	
Due to disease	3 (4.2)
Due to technical problem	9 (12.7)
12-months mortality	21 (29.6)
Dietician sessions: Median (25th-75th)	2 (1-3)
Nutritional consultation	
No	25 (35.2)
By oncologist	26 (36.6)
By dietician	20 (28.2)

**Table 3:** Components of Dietary Intake of Participants

Variables	T1 (Baseline)	T2	Change #	T3	Change ##
		(After CRT)	(T2-T1)	(Late evaluation) (n=32)	(T3-T1)
Energy Intake					
(kcal/day)	1064.4±620	958.8±527	-105.6±735	1448±584*	245.2±664
(Kcal/kgW.d)	19.5±11	18.3±11	-1.2±14	27.8±13*	6.4±14
Protein Intake					
(gr/day)	30.8±21	34.2± 21	3.4±28	47.5±27*	11.5±28
(g/kgWday)	0.56±0.4	0.66±0.5	0.10±0.6	0.93±0.6*	0.31±0.6
Carbohydrate					
(g/ day)	147.2±74	128.6±65	-18.6±90	195.2±95	32.5±104
Fat (g/day)	42.6±42	35.2±26	-7.3±48	56.8±28	7.7±47
Fiber (g/d)	7.9±8	6.1±5	-1.7±10	10.2±8	1.6±10
Iron (mg/day)	4.3±4	4.4±5	0.1±6	6.4±5	1.8±6
Zinc (mg/day)	3.5±3	3.9±3	0.4±4	6.7±8	2.6±8
Calcium (mg/day)	482.0±303	637.0±502	155.0±601	753.7±461*	206.7±467
Vitamin C (mg/day)	121.1±102	124.2±204	3.2±190	147.6±145	32.3±142
Vitamin E (mg/day)	4.4±7	3.1±3	-1.3±7	8.9±17	3.6±19
Vitamin B6 (µg/day)	1.0±1	1.4±3	0.5±3	1.4±1	0.3±2
Vitamin D (IU/day)	19.6±24	19.7±30	0.1±37	35.3±49	12.0±56.6

CRT, Chemoradiation; All values are means± standard deviations; p-value obtained from paired-samples t-test; * p<0.05; Late evaluation is considered 4-6 weeks after chemoradiation; #, Changes between baseline and end of chemoradiation; ##, Changes between baseline and 4-6 weeks after chemoradiation

**Table 4:** PG-SGA Score, Anthropometric Indices, Physical Performance, and Biochemical and Hematological Tests of Participants

Variables	T1 (Baseline)	T2 (After CRT) (n=48)	Change (T2-T1) #	*P*- value^a^#	T3 (Late evaluation) (n=32)	Change (T3-T1) ##	*P*- value^a^##
PG-SGA score	13.0±6	14.0±6	1.0±7	0.332	9.3±6	-3.4±8	0.016
Weight (kg)	57.02±16.9	54.60±16.5	-2.42±2.4	<0.001	55.24±13.6	-2.69±3.8	<0.001
MUAC (cm)	26.68±4.9	25.42±5.1	-1.27±1.4	<0.001	25.26±4.9	-1.49±1.7	<0.001
Fat Mass (%)	24.11±11.8	22.8±12.5	-1.5±3.5	<0.001	21.17±12.3	-3.15±4.4	0.001
FFMI (Kg/M^2^)	16.87±2.4	16.47±2.6	-0.50±1.1	0.006	17.0±2.3	-0.05±1.2	0.823
Hand grip strength (kg)	43.2±19	36.1±20	-6.2±9	<0.001	36.4±18	-4.8±9	0.01
Karnofsky	81.2±15	75.8±18	-5.4±19	0.052	83.3±18	1.9±19	0.582
WBC (cells/mm^3^)	7286±2880	3593±3650	-3693±4190	<0.001	4930±1450	-2371±3000	<0.001
TLC (cells/mm^3^)	1979±770	648±490	-1329±840	<0.001	1687±600	-220±670	0.083
Hemoglobin (cells/mm^3^)	12.71±1.8	11.49±1.6	-1.22±1.5	<0.001	12.10±3.8	-0.78±3.6	0.234
RDW (%)	14.15±1.7	16.1±2.5	1.94±1.7	<0.001	17.16±2.4	3.12±2.5	<0.001
Platelets (*1000) (/mm^3^)	268±70	185± 70	-83.2±73	<0.001	248±93	-13.8±80	0.346
CRP (mg/dl)	16.71±23.3	18.15±18.3	1.44±21	0.675	8.18±8.3	-9.63±24.0	0.033
Total protein (g/dL)	7.52±0.6	6.85±0.6	-0.67±0.8	<0.001	7.18±0.7	-0.40±0.8	0.007
Albumin (g/dL)	4.02±0.4	3.57±0.5	-0.45±0.49	<0.001	3.8±0.4	-0.16±0.6	0.115

CRT, Chemoradiation; PG-SGA, Patient-Generated - Subjective Global Assessment; MUAC, mid-upper arm circumference; FFMI, fat-free mass index; VAS, visual analogue scale; WBC, white blood cells; TLC, Total lymphocyte count; RDW, red blood cell distribution width; CRP, C-reactive protein; All values are means± standard deviations; Late evaluation is considered 4-6 weeks after chemoradiation; a p-value obtained from paired-samples t-test; #, Changes between baseline and end of chemoradiation; ##, Changes between baseline and 4-6 weeks after chemoradiation

**Table 5:** Nutritional Status Related Factor Associated with 12-Months Mortality

Variables	12 months survived patients (n=50)	12 months deceased patients (n=21)	*P*-value
Age (year)	66.3±11	65.5±14	0.949^a^
Gender			0.692^b^
Men	24 (48)	9 (43)	
Women	26 (52)	12(57)	
Living place			0.023 ^b^
Rural	26 (52)	17 (81)	
Urban	24 (48)	4 (19)	
Addiction	9 (18)	11 (52)	0.013 ^b^
Smoking	13 (26)	5 (24)	0.846 ^b^
Economic status			0.033 ^b^
Low	33 (66)	19 (90)	
Moderate or high	17 (34)	2 (10)	
PG-SGA score before CRT	12.4±5	16.5±6	0.005 ^a^
Dysphagia# before CRT	23 (46)	15 (71)	0.05
BMI after CRT (kg/m^2^)	22.9±6.1	18.63±4.9	0.022 ^a^
BMI<18.5 kg/m^2^ before CRT	13 (26)	11 (52)	0.032 ^a^
Weight loss before CRT			0.013^b^
Mild	30 (60)	5 (24)	
Moderate	14 (28)	9 (43)	
Severe	6 (12)	7 (33)	
MUAC before CRT (cm)	26.9±5	23.8±5	0.013 ^a^
Fat Mass percentage after CRT	23.4±11	20.7±13	0.413 ^a^
FFMI (Kg/M^2^)			
Before	16.67±2.4	16.02±2.7	0.359 ^a^
After CRT	16.71±2.8	15.7±1.6	0.321 ^a^
Hand grip strength (kg) after CRT	35.7±20	37±18	0.841 ^a^
Karnofsky before CRT	82±13	72±18	0.012 ^a^
CRP (mg/dl)			
before CRT	11.5±12	20.3±29	0.084 ^a^
after CRT	16.5±18	22.1±19	0.404 ^a^
Total protein (g/dL)	7.49±0.6	7.28±0.8	0.211 ^a^
Albumin (g/dL)	3.94±0.5	3.83±0.6	0.402 ^a^
RDW			
Before CRT	14.0±1.7	14.1±1.3	0.142 ^a^
After CRT	16.27±2.8	15.63±1.1	0.450 ^a^

CRT, Chemoradiation; PG-SGA, Patient-Generated - Subjective Global Assessment; BMI, body mass index; MUAC, mid-upper arm circumference; FFMI, fat-free mass index; RDW, red blood cell distribution width; CRP, C-reactive protein; Data has reported n (percentage) or mean± standard deviation; #Dysphagia which resulted in reduced intake of patients; ^a ^P-value obtained from paired-samples t-test; ^b^ P-value obtained from chi square test.

## Discussion

This study showed nutritional status deterioration, insufficient dietary intake, and significant weight loss and high PG-SGA score, and reduced physical performance during chemoradiation and even 4 to 6 weeks post-treatment; although this is a favorably selected series because clearly malnourished patients were excluded.

In this study, with a mean PG-SGA score of 13.1±6 at the beginning of treatment, 79.8% of patients had a PG-SGA score > 8 requiring nutritional intervention. The mean PG-SGA score remained high after CRT (14.0±6) and 4 to 6 weeks post-treatment treatment (9.3±6). In addition, 55.7% of patients did not meet their energy requirements and 89.8% of patients consumed inadequate protein at the beginning of treatments. The energy and protein intake inadequacy persisted during CRT. 

The deterioration of nutritional indices during treatment can be explained by acute side effects of radiation therapy and chemotherapy such as esophagitis and nausea. However, 4 to 6 weeks post-treatment, energy and protein inadequacy were found in 27.3% and 72.7% of patients showing a partial improvement probably due to partial relief of dysphagia and anorexia, and yet, with high rate of malnutrition, in our survey study during CRT and follow-up, only 20 participants (28.2%) referred to the nutritionist, and 26 patients (36.6%) had nutritional intervention by their oncologists including oral nutrition supplementation in 16 participants and the other 10 patients received some intravenous intralipid and amino-acid serums. Only 2 patients had ONS supplementation more than 200 kcal/day during treatment. None of the partial parenteral nutrition administrated by oncologists meet the patients’ energy and protein needs. These results emphasize on the necessity for a dietary consultation and intervention by a dietician for most patients.

Micronutrients intakes were also lower than dietary reference intakes at baseline and after CRT and even 4 to 6 weeks after treatment. Micronutrient sufficiency impacts on the patient’s physical and mental quality of life and the efficiency of treatment, and their deficiency leads to immunocompetence impairment, increases the risk of complications (Grober et al., 2016). It is also detected in other cancers (Guren et al., 2006) and proper supplementation could improve patients nutritional status and wellbeing (Grober et al., 2016; Arends et al., 2017).

Esophageal cancer may cause severe malnutrition due to dysphagia and invasive nature of the disease. Zhang (2014) reported a PG-SGA score 11.07± 4.03 for esophageal cancer which was the highest among gastrointestinal cancers (Zhang et al., 2014). Also, it has been shown that pretreatment nutritional status of esophageal cancer patients as a significant predictor of overall survival (hazard ratio= 12.45). Moreover, nutritional intervention improved survival only if provided at baseline and before CRT (Cox et al., 2016). 

In this study, 18% and 32% of participants had severe and moderate weight loss before treatment. We found a significant weight loss during CRT in our patients. Di Fiore (2006) showed that weight loss of more than 10 % had a significant negative impact on survival in multivariable analysis (Di Fiore et al., 2006). We observed a significant reduced muscle mass, hand grip strength as well. Chen (2011) acclaimed that low muscle strength in esophageal cancer patients is associated with more mortality rate, ICU stay and complications. They also introduced hand grip strength measurement as a non-invasive, simple and cheap with high predictive value tool for routine preoperative evaluation in these patients(Chen et al., 2011). 

In this study, nutritional related test including WBC, total lymphocyte count (TLC), RDW, albumin, total protein reduced significantly during chemoradiation. Albumin has been reported as an independent predictive factor of complete response to CRT in esophageal cancer patients (Fiore et al., 2007). Elevated RDW predicts poor prognosis in patients with oral SCC and hepatocellular carcinoma. (Zhu et al., 2017; Ge et al., 2018).

In present study, the twelve-months mortality was significantly associated with PG-SGA score before CRT, BMI after CRT, BMI<18.5 before CRT, weight loss intensity before CRT, MUAC before CRT, Karnofsky physical performance before CRT. Fiore et al. studied the nutritional status parameters at baseline and their relation with survival in advanced esophageal cancer patients (Fiore et al., 2007). They found a significant association of survival with lower dysphagia score, BMI>18. In contrast to our finding, Chen (2011) showed an association between hand grip strength and 12-month mortality. Hand grip strength was measured before surgery and due to long term of malnutrition after malignancy manifestations, the difference was more than this study (Chen et al., 2011). In this study, we did not find any association between 12- month mortality and pretreatment serum albumin or total protein probably because almost all patients had normal serum total protein or albumin levels. Living in rural area, addiction, and economic status was associated with higher one-year mortality rate. These results could be due to poorer nutritional status in these groups. 

In patients with esophageal cancer, weight loss, intake energy and protein intake can be attributed to the nature of the advanced malignant disease, anorexia and dysphagia. The nutritional status can be worsened during chem-radiotherapy due to esophagitis, nausea and altered taste. In these patients, the altered adequate nutritional intake can be managed at least partially by dietary modification and nutritional consultation. Therefore, consultation with an experienced nutritionist is crucial to improve nutritional status by nutritional intervention and potentially enhance survival. 

The strength of our study was performing comprehensive nutritional assessment at the beginning, after and 4 to 6 weeks post-treatment with single researcher. 

The most important limitation of our study was the lack of nutritional assessment during CRT; considering that treatment modalities including radiotherapy and chemotherapy can worsen nutritional status and subsequent complications in short term.

For future studies, we propose that changes in nutritional status be evaluated during treatment, due to great effect of chemo-radiation on cancer patients’ nutrition.

In conclusion, our study showed the prevalence of malnutrition in esophageal cancer patients is very high even at the beginning of malignancy management and deteriorated during CRT and remained 4 to 6 weeks after oncologic treatments. Early screening of the nutritional status of these patients, early referral to dieticians, and effective nutritional interventions are necessary and life-saving.
